# Long-distance dispersal to oceanic islands: success of plants with multiple diaspore specializations

**DOI:** 10.1093/aobpla/plv073

**Published:** 2015-07-14

**Authors:** Pablo Vargas, Yurena Arjona, Manuel Nogales, Ruben H. Heleno

**Affiliations:** 1 Real Jardín Botánico de Madrid (RJB-CSIC), 28014 Madrid, Spain; 2 Island Ecology and Evolution Research Group (IPNA-CSIC), 38206 La Laguna, Tenerife, Canary Islands, Spain; 3 Centre for Functional Ecology, Department of Life Sciences, University of Coimbra, 3000-456 Coimbra, Portugal

**Keywords:** Anemochorous, diplochorous traits, endozoochorous, epizoochorous, insular colonization, thalassochorous

## Abstract

A great number of scientific papers claim that angiosperm diversification is manifested by an ample differentiation of diaspore traits favouring long-distance seed dispersal. Oceanic islands offer an ideal framework to test whether the acquisition of multiple sets of diaspore traits (syndromes) by a single species results in a wider geographic distribution. To this end, we performed floristic and syndrome analyses and found that diplochorous species (two syndromes) are overrepresented in the recipient flora of the Azores in contrast to that of mainland Europe, but not to mainland Portugal. An additional analysis of inter-island colonization showed a general trend of a higher number of islands colonized by species with a single syndrome (monochorous) and two syndromes than species with no syndrome (unspecialized). Nevertheless, statistical significance for differences in colonization is meagre in some cases, partially due to the low proportion of diplochorous species in Europe (244 of ∼10 000 species), mainland Portugal (89 of 2294 species), and the Azores (9 of 148 species), Canaries (17 of 387 lowland species) and Galápagos (18 of 313 lowland species). Contrary to expectations, this first study shows only a very marginal advantage for long-distance dispersal of species bearing multiple syndromes.

## Abstract (precis)

After more than 180 million years of evolution, angiosperms have acquired multiple fruit and seed types. Oceanic islands offer an ideal framework to test the advantage of bearing multiple sets of diaspore traits by a single species using its geographic distribution. Contrast analyses between the floras of Europe (Portugal) and Azores revealed a meager advantage for species with two sets of diasporas vs one set or none. A general trend of a higher number of islands colonized by species with a single set and two sets than species with no traits related to long-distance dispersal (unspecialized) was found.

## Introduction

The evolutionary acquisition of fruits by angiosperms offered the opportunity to have one more dispersal-related structure (the fruit) subject to modification and natural selection (Vargas 2014). In particular, infructescences, fruits and seeds (reproductive diaspores) have been evolving specific traits involved in specializations that favour long-distance dispersal (hereafter LDD) and thus higher colonization success (Valcárcel and Vargas 2014). After more than 180 million years of evolution, angiosperms have spawned multiple evolutionary avenues, including a great diversity of fruit and seed types. This is interpreted as a result of selection on diaspore traits assisting the colonization of new territories and environments (Valcárcel and Vargas 2014). But, how effective have LDD syndromes actually been? The comparison of the floras of Europe and the Azores shows that some sets of diaspore traits (syndromes), chiefly specializations for floatation and survival in seawater (thalassochorous traits), appear to have been favoured in the colonization of the archipelago (Heleno and Vargas 2015). However, Europe (continental source) shows a majority of species with unspecialized diaspores (∼63 %) that parallels the poor specialization (∼63 %) of the flora of the Azores islands (recipient flora) (Heleno and Vargas 2015). Acquisition of diaspore and floral syndromes has been very dynamic in the course of evolution, and thus any flora constitutes a complex assemblage of specialized and generalized species (Whitney 2009). Most floras have some species adapted towards accumulating diaspore traits that can facilitate dispersal by more than one mechanism; for example, palatable fleshy fruits that can float and survive after immersion in saltwater can be dispersed internally by animals and/or by oceanic currents (Ridley 1930). This type of species is named diplochorous (see Vander Wall and Longland 2004). The question remains as to whether diaspores with multiple specializations (more than one set of diaspore traits) have been particularly favoured for LDD. Indeed, the theory of island biogeography predicts that oceanic archipelagos are disharmonic inasmuch as they represent a sample of the mainland diaspore pool, i.e. dispersal (diaspore specializations) and further establishment (habitat availability) act as filters to colonization (Whittaker and Fernández-Palacios 2007). Therefore, our working hypothesis is that plants with multiple diaspore specializations have been particularly favoured in the process of island colonization.

Diplochory has been defined as seed dispersal by a sequence of two or more steps or phases, each involving a different dispersal agent (Vander Wall and Longland 2004). Nevertheless, it is extremely difficult to document multiple dispersal events of new colonizer species on islands, and virtually impossible to reconstruct the colonization processes over long periods of time (millions of years). An alternative approach is to evaluate whether species having multiple syndromes (i.e. more than one set of traits related to wind, sea currents or diaspore dispersal by animals—both internally and externally) have been particularly favoured for LDD over species with a single or no LDD syndrome. Although distribution frequencies of single LDD syndromes between floras of distant territories, such as Tasmania–New Zealand (Jordan 2001) and Europe–Azores (Heleno and Vargas 2015), have already been analysed, the additional value of bearing multiple syndromes on a single species remains unexplored. Species spectra of insular floras offer different datasets to contrast the colonization success of species bearing no, one, two or more diaspore syndromes. Comparative analyses of LDD syndrome distributions provide a general framework to test this and other explicit evolutionary hypotheses about dispersal traits potentially favoured for island colonization within the theory of island biogeography (Heleno and Vargas 2015). For instance, a long-standing hypothesis posited for Hawaiian plants proposed loss of dispersability during the process of evolution on islands (Carlquist 1966). The most suitable approach to test dispersability changes is the phylogenetic method because it evaluates shifts of diaspore syndromes related to LDD (reconstruction of ancestral characters) (Vargas 2007). Alternatively, species spectra that contrast traits of endemic vs. indigenous (Vargas *et al*. 2014) and congeneric species pairs (Vazačová and Münzbergová 2014) have already been used to test loss of dispersability.

Oceanic islands emerge lifeless from the sea floor and receive all their species by LDD. That is why oceanic archipelagos provide an ideal spatio-temporal system in which to analyse plant dispersal traits related to current distributions of species across islands. The present study evaluates the success of species with multiple dispersal syndromes in island colonization, by analysing the floras of the European continent (including mainland Portugal) and the Azores. In addition, the success of multiple dispersal syndromes in inter-island colonization is analysed for the floras of the Azores, Canaries and Galápagos. Specific questions are as follows. (i) How are multiple sets of LDD traits distributed within each species and within each flora? (ii) Are multiple dispersal syndromes overrepresented in the recipient flora of the Azores, relative to the source floras of Europe and mainland Portugal? (iii) How well distributed are species with no, one and multiple dispersal syndromes within each archipelago? (iv) Is there evidence of loss or acquisition of multiple dispersal syndromes during speciation on islands?

## Methods

### Flora of Europe and Azores

Contrast analyses were performed on full spectra of LDD syndromes from the native Azorean flora (recipient) and those of the source floras of Europe and mainland Portugal (see Heleno and Vargas 2015). These analyses are largely complementary because the European flora includes the closest related continental flora available for colonization of the Azores, while the subset formed by the mainland Portuguese flora represents a more comparable territory in terms of habitat similarity, notably maximum elevation, latitude range, coastal length and historical climate (see Patiño *et al*. 2015). The lists of plant species native to Europe (10 792 species) and mainland Portugal (∼2294 species) were retrieved from Flora Europaea (Tutin *et al*. 1980) (see Heleno and Vargas 2015); the Azorean flora, comprising 148 native species, was retrieved from Schaefer *et al*. (2011). To contrast the proportions of species syndromes between the Azores and the two mainland floras, we excluded endemic and introduced species from the analysis, using only shared native species.

### Archipelago floras

The relative importance of LDD syndromes within the floras of the Galápagos (Pacific Ocean) and the Azores (North Atlantic Ocean) has been recently evaluated (Heleno and Vargas 2015; Vargas *et al*. 2014). One more North Atlantic archipelago (the Canary Islands) has partially been analysed for syndrome categorization (Arjona *et al*. 2015) and is herein used to evaluate the effect of multiple dispersal syndromes on island LDD and colonization. The species used in this study share similar habitats (typically lowland habitats) that occur on all the islands of each archipelago (Aranda *et al*. 2014; Vargas *et al*. 2014). In other words, we excluded medium- and high-altitude species from the analysis of the Galápagos and the Canaries, as their habitats are not present on all islands; thus inter-island connectivity could be tested because all the islands share similar lowland conditions for establishment. All the Azores species have been included because of their low number; altitudinal zonation is not critical there and habitat limitation is an unlikely barrier to colonization. As a result, the three archipelagos display different numbers of species: the Azores (148 native species, Schaefer *et al*. 2011), Canary Islands (387 lowland of 703 native species, Acebes Girovés *et al.* 2010) and Galápagos (313 lowland of 403 native species, Vargas *et al*. 2014). The distribution of species across islands of each archipelago (i.e. number of islands where present) is used as a proxy for diaspore LDD capacity. Therefore, the study contrasts the distribution of species across islands under similar ecological conditions and considering two or more dispersal syndromes (vs. the distribution of species with a single syndrome or none). Each archipelago was initially analysed independently, after which general patterns were then compared among islands.

### Number of islands

The three archipelagos included in this study have different numbers of large islands (>10 km^2^): the Azores (9), Canaries (9) and Galápagos (12). However, fluctuating sea level (eustasy) and volcanic activity imply that some present islands might have been connected by land bridges in the past (Ali and Aitchison 2014). Thus, colonization across long-standing islands is better studied based on the number of islands that have been isolated since emergence (palaeo-islands). Accordingly, we used the number of palaeo-islands pre-dating the last glaciations for each archipelago, namely seven in the Galápagos (Ali and Aitchison 2014), eight in the Azores (Rijsdijk *et al*. 2014) and six in the Canaries (Fernández-Palacios *et al*. 2011).

### Diaspore traits and syndrome assignment

Long-distance dispersal is herein understood in a biogeographical sense, i.e. plant connections between the mainland and the Azores and among islands within the same archipelago (Azores, Canaries, Galápagos). We classified diaspores into five classes according to the presence/absence of specialized morphological traits favourable to particular LDD vectors: anemochory (wings, plumes or hairs promoting dispersal by wind), thalassochory (floatation and survival favouring dispersal by oceanic currents), endozoochory (nutritive tissues promoting internal dispersal by animals), epizoochory (hooks, hairs or adhesive substances aiding external dispersal by animals) and unspecialized (no LDD traits). For a detailed guide to syndrome categorization see Appendices in Heleno and Vargas (2015). As we intentionally do not consider information regarding ‘actual dispersal’ for syndrome assignment, the categorization includes four sets of diaspore traits that potentially provide an evolutionary advantage for LDD, namely anemochorous, thalassochorous, endozoochorous and epizoochorous traits (see Van der Pijl 1982). Therefore, as an important difference regarding other studies, we did not analyse actual dispersal for inter-island colonization (i.e. actual vectors) or categorization into specific and often fuzzy dispersal mechanisms (e.g. mud dispersal; see Nogales *et al*. 2012). Alternatively, our approach tests the likely success of particular morphologies (diaspore specializations) acquired in the evolutionary history of angiosperms. Previous studies have already implemented this syndrome approach for plants from the Galápagos (Vargas *et al*. 2014) and Azores (Heleno and Vargas 2015). The same analysis has been performed for part of the flora of the Canaries (Arjona *et al*. 2015).

### Multiple syndromes in single species

Combining two means of diaspore dispersal can increase the probability of seed dispersal reaching suitable habitats and reducing seed mortality (Vander Wall and Longland 2004; Valcárcel and Vargas 2014). This typically includes multiple adaptations of different plant dispersal units (seeds, fruits or infructescences) related to the four LDD syndromes; for instance *Astydamia latifolia* of the Canary Islands has a winged fruit (anemochorous) with seeds that survive a long time in sea water (thalassochorous) (Y. Arjona *et al*. unpubl. data). The two syndromes can be displayed on different diaspore parts, as in the case of *A. latifolia* (above), or on the same diaspore part, as in *Corema alba* from the Azores that displays fleshy fruits promoting both endozoochory and thalassochory (C. F. Esteves *et al*. unpubl. data). In some other cases the same species can have intra-individual variation in the way that the same plant part has two different diaspore types; for instance, Asteraceae and some other families have inflorescence heterocarpy—that is two types of fruits such as adhesive achenes on the capitulum periphery and plumed achenes in the capitulum centre (Sorensen 1986).

Irrespective of the origin of multiple diaspore adaptations within a single species, three groups of species were considered: with unspecialized diaspores, with one syndrome (monochorous) and with two or more syndromes (multichorous). Datasets for the five floras (Europe, Portugal, Azores, Canaries and Galápagos) were taken from our previous studies and reanalysed after careful examination of one or more syndromes on single species. We also studied multichorous species to find out if they could be grouped into diplochorous (two-syndrome) and triplochorous (three-syndrome) groups. Accordingly, this classification also suitably incorporates the evolutionary advantage of seed specializations that potentially favour travelling by multiple, consecutive dispersal vectors.

### Statistical analyses

To evaluate if species with more than one LDD syndrome were particularly favoured in the colonization of the Azores from mainland diaspores, we used a contingency analysis. A likelihood ratio test (*G* test) was used to compare the proportion of insular species with multichorous diaspores vs. those in continental Europe and continental Portugal. To find any signal of loss of dispersability, proportions of diaspore specializations from non-endemic natives (expressing speciation within the continent) and archipelago endemics (speciation on islands) were also analysed using a likelihood ratio test.

The effect of having no, one or multiple LDD syndromes (categorical predictor) on the distribution of plant species within each archipelago was evaluated with generalized linear models, considering the number of palaeo-islands (Poisson distributed error) as the dependent variable. When an effect was detected, multiple comparisons between each flora type (diaspores with unspecialized, monochorous and multichorous traits) were performed by a Tukey post hoc test. In order to look for an overall effect of unspecialized, monochorous and diplochorous diaspores on plant distribution across all datasets, we used a generalized linear mixed-effects model, including ‘archipelago’ as a random variable and assuming a Poisson distribution for the number of palaeo-islands. This test was followed by a Tukey multiple comparisons test. All analyses were performed using the packages Base, lme4, multcomp and Deducer in R v. 3.1.0 (R Development Core Team 2012).

## Results

The results shown here are based on the codification of diaspore traits into LDD syndromes in ∼11 000 angiosperm species from the floras of Galápagos, Azores, Canaries and continental Europe, including mainland Portugal.

### Distribution of multichorous traits between floras

**Figure 1.**
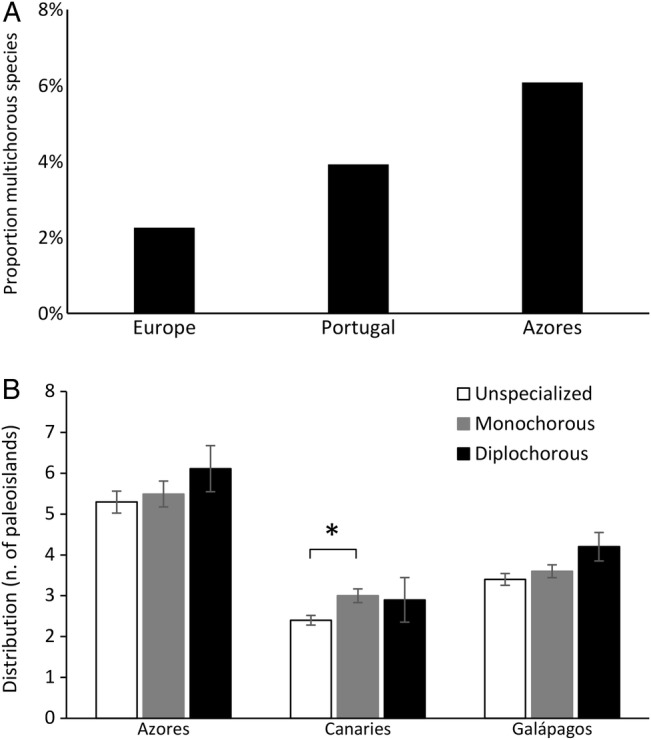
(A) Proportion of multichorous (diplochorous) species in mainland (Europe, Portugal) and the Azores. (B) Mean distribution of plant species with one, two and no LDD syndromes (monochorous, diplochorous and unspecialized, respectively) within the palaeo-islands of the Azores, Canaries and Galápagos. Error bars indicate the standard error of the mean. Significant differences (to *α* = 0.05) are marked with an asterisk.

None of the species analysed had more than three syndromes and even three syndromes were identified in only five species (<0.01 % of the species screened). These five species shared traits related to anemochory, epizoochory and thalassochory, and were only present in mainland Europe: two *Limonium* (*L. lobatum* and *L. sinuatum*), two *Vulpia* (*V. alopecuros* and *V. fasciculata*) and one *Armeria* (*A. maritima*). We did not find any insular species with three or more sets of LDD syndromes. Species with two syndromes are much more frequent than those with three syndromes and represent a low proportion of the floras of Europe (244 of ∼10 000 species; 2.4 %) and mainland Portugal (89 of 2294 species; 3.9 %) (Fig. 1A). Insular floras also displayed a low representation of diplochorous traits (considering species with potential habitat on all islands—see Methods): Azores (9 of 148 species; 6.1 %), Canaries (17 of 387 lowland species; 4.4 %) and Galápagos (18 of 313 lowland species; 5.8 %).

As a whole, the three archipelagos displayed 44 species with two syndromes, with approximately half of them (21 species; 48 %) having both anemochorous and thalassochorous traits (Table 1). In the flora of Europe, the highest proportions are found when combining anemochorous and epizoochorous (68.5 %), followed by anemochorous and thalassochorous (23.0 %) traits. Only in Europe we found three species (*Adoxa moschatellina*, *Viscum album* and *Viscum cruciatum*) with both epizoochorous and endozoochorous traits, and one species (*Corema alba*) with both thalassochorous and endozoochorous traits. The only syndrome pair not found in any of the studied floras is that including both anemochorous and endozoochorous traits (Table 1C).

**Table 1.** Frequency of two shared LDD syndromes on the same species of the following floras: (A) the Azores, mainland Portugal and Europe; (B) the Azores, Canaries and Galápagos and (C) the three archipelagos (altogether). The three archipelagos only have species with diplochorous traits, while Europe and Portugal also include a few cases of triplochorous species.

	Endozoochorous			Epizoochorous			Thalassochorous		
	Azores	Portugal	Europe	Azores	Portugal	Europe	Azores	Portugal	Europe
(A)									
Epizoochorous	0	2	3	–	–	–	–	–	–
Thalasochorous	1	1	1	2	7	18	–	–	–
Anemochorous	0	0	0	4	57	161	2	16	54
	Endozoochorous			Epizoochorous			Thalasochorous		
	Azores	Canaries	Galápagos	Azores	Canaries	Galápagos	Azores	Canaries	Galápagos
(B)									
Epizoochorous	0	0	3	–	–	–	–	–	–
Thalassochorous	1	0	7	2	1	1	–	–	–
Anemochorous	0	0	0	4	3	1	2	13	6
	Endozoochorous	Epizoochorous	Thalassochorous						
(C)									
Epizoochorous	3	–	–						
Thalassochorous	8	4	–						
Anemochorous	0	8	21						

### Diplochorous traits within and among islands

The proportion of species with more than one LDD syndrome is significantly higher in the Azores than in the whole flora of Europe (*G* = 6.19, df = 1, *P* = 0.013) and also higher, but not significantly so, than that of Portugal (*G* = 1.37, df = 1, *P* = 0.248), which is a more comparable territory.

Overall, there is a weak tendency for species bearing more LDD syndromes to occur on more islands (Fig. 1B, Table 2). Nevertheless, this tendency is non-significant (Azores *χ*^2^ = 1.09, df = 2, *P* = 0.59; Galápagos *χ*^2^ = 2.85, df = 2, *P* = 0.25; Table 3) with the exception of a broader distribution for species with one syndrome than species with no syndromes in the Canary Islands (Canaries *χ*^2^ = 12.35, df = 2, *P* = 0.0022; Tukey’s test *z* = −3.45, *P* = 0.0016) (Tables 3 and 4). Figure 1 also shows that the Azores archipelago clearly harbours a wider distribution of species, irrespective of the presence or absence of syndromes, followed by Galápagos and then the Canaries. When considering the three archipelagos together, plant distribution is significantly affected by the presence and number of LDD syndromes (*χ*^2^ = 10.82, df = 4, *P* = 0.0045). Post hoc comparisons revealed that the distribution of plants with one and two syndromes was higher than that of plants with unspecialized diaspores (*χ*^2^ = −2.76, *P* = 0.014, and *z* = −2.40, *P* = 0.014, respectively); however, there was no difference between the distribution of species with one and multiple syndromes (*z* = 1.04, *P* = 0.539; Table 5).

**Table 2.** Distribution of species displaying multiple (multichorous), single (monochorous) and unspecialized traits related to LDD within the Azores, Canaries and the Galápagos, measured as the mean number of palaeo-islands.

Diaspore type	Azores	Canaries	Galapagos
Total number of palaeo-islands (potential distribution)	8	6	7
Unspecialized	5.3 ± 2.4	2.4 ± 1.7	3.4 ± 1.7
Monochorous	5.5 ± 2.4	3.0 ± 2.1	3.6 ± 1.8
Multichorous	6.1 ± 1.6	2.9 ± 2.2	4.2 ± 1.4

**Table 3.** ANOVA table for the three generalized linear models explaining the distribution of plant species within Azores, Canaries and Galápagos (number of palaeo-islands where each species occurs; Poisson distributed) by the number of dispersal syndromes present on their diaspores (categorical variable: unspecialized; one dispersal syndrome; two or more (multiple) dispersal syndromes). One model was constructed for each archipelago.

		df	Deviance	Residual df	Residual deviance	*χ*^2^	*P* value
Azores	Null			147	180.04		
	Number of syndromes	2	1.062	145	178.98	1.092	0.5881
Canaries	Null			386	504.73		
	Number of syndromes	2	12.253	384	495.48	12.352	0.00218
Galápagos	Null			298	273.56		
	Number of syndromes	2	2.741	296	270.82	2.859	0.254

**Table 4.** Summary information of the multiple comparisons performed with the Tukey post hoc test, exploring differences on plant distribution across the Canaries palaeo-islands, according to the number of dispersal syndromes present on their diaspores (categorical variable: 0, unspecialized; 1, one dispersal syndrome; ≥2, two or more dispersal syndromes).

	Estimate	SE	*Z* value	*P* value
≥2 vs. 1 syndrome	−0.031	0.149	−0.207	0.97546
0 vs. 1 syndrome	−0.220	0.064	−3.455	0.00163
0 vs. ≥2 syndromes	−0.148	0.150	−1.280	0.38932

**Table 5.** Summary information of the multiple comparisons performed with the Tukey post hoc test, exploring differences on plant distribution across the palaeo-islands of three archipelagos (Azores, Canaries and Galapagos, as random factor) taken together, according to the number of dispersal syndromes present on plant diaspores (categorical variable: 0, unspecialized; 1, one dispersal syndrome; ≥2, multiple dispersal syndromes). *Significant differences at α = 0.05.

	Estimate	SE	*Z* value	Adj. Pr(>|*z*|)
≥2 vs. 1 syndrome	0.082	0.080	1.039	0.5386
0 vs. 1 syndrome	−0.106	0.038	−2.761	0.0144*
0 vs. ≥2 syndromes	−0.188	0.078	−2.396	0.0401*

### Diplochorous traits in endemic vs. indigenous species

We failed to find significant differences between the proportion of plants with diplochorous traits between the endemic and non-endemic native flora of the Azores (*G* = 0.29, df = 1, *P* = 0.59), Canaries (*G* = 0.88, df = 1, *P* = 0.35) and Galápagos (*G* = 0.54, df = 1, *P* = 0.46). Accordingly, island species displayed neither gain nor loss of diaspore traits related to LDD.

## Discussion

Diaspore specialization of European angiosperms for LDD appears to be poor, inasmuch as the majority of the species from the two insular and two mainland floras show a high level of unspecialized diaspores (54–67 %) (see Heleno and Vargas 2015; Arjona *et al*. 2015). A similar figure was found for the flora of Galápagos (55.6 %; Vargas *et al*. 2014). These unspecialized plants were successful long-distance dispersers despite the apparent disadvantage of lacking specialized adaptations for LDD. Indeed, we know from landscape studies that particular sets of diaspore traits can increase both dispersal success and seedling establishment (Schupp *et al*. 2010). To the best of our knowledge, the hypothesis that plants displaying multiple sets of diaspore traits adapted for abiotic (sea, wind) and biotic (animal) LDD dispersal result in greater success in colonizing islands has, however, not been tested yet.

### Multiple LDD syndromes in continental and insular floras

In a recent study, Heleno and Vargas (2015) found that only plants displaying diaspores adapted for sea dispersal (thalassochory) showed evidence of overrepresentation in the flora of Azores with respect to the mainland European and Portuguese floras. Here we show that diplochorous traits are also overrepresented in the Azores with respect to the flora of mainland Europe (but not to that of Portugal). Given that bioclimatic conditions (habitat similarity, maximum elevation, latitude range, coastal length, climate) of the flora of the Azores rather parallel conditions of mainland Portugal (see Heleno and Vargas 2015; Patiño *et al*. 2015), the contrast between Europe and Azores should be considered with caution.

Certain combinations of two syndromes are predominant in temperate floras. Indeed, the majority of the diplochorous species in the floras of the Azores, Canaries and mainland Europe have anemochorous coupled with epizoochorous or thalassochorous traits. The predominance of these two pairs of syndromes in the three temperate floras leads us to hypothesize a primary role of either evolutionary constraints of angiosperms (as a whole) in the process of acquisition of co-occurring morphologies or the existence of ecological conditions favouring certain syndrome pairs depending on biogeographic areas. It is a fact that diplochorous traits in the tropical flora of Galápagos result from endozoochorous coupled with epizoochorous or thalassochorous traits, which are very rare in temperate floras (only four cases in Europe). This favours the hypothesis of differential adaptation of fleshy fruits in tropical areas (Ridley 1930; Moles *et al*. 2007). Indeed, in woody species, endozoochorous seeds are more frequent in neo- and palaeotropical (>70 %) than in temperate forests (<44 %) (Jordano 2014). The association between syndrome combination and latitude needs to be further explored given the scarce knowledge of dispersal syndromes in some biogeographic areas (see Fig. 3 in Moles *et al*. 2007).

### Inter-island colonization within the Azores, Canaries and Galápagos

There is a low number of species displaying multiple syndromes in the floras of Azores (9 species), Canaries (17 species) and Galápagos (18 species). Nevertheless, an analysis of all diplochorous species from the three archipelagos (44 species) shows statistical significance for a general pattern of a higher number of islands colonized by species with two syndromes (diplochorous) than species with no syndromes (unspecialized). The same is true for a single syndrome (monochorous) vs. unspecialized species from the three archipelagos. Overall, we confirmed the expected advantage for intra-island colonization of species with either one or two syndromes when compared with unspecialized species. However, we failed to detect any measurable advantage of species bearing any combination of two syndromes when compared with monochorous species.

Vander Wall and Longland (2005) proposed two models of evolutionary transition for species with multiple syndromes: co-occurrence of either two competing or two sequential modes of primary dispersal over evolutionary periods. To the best of our knowledge, there is no study that tests these evolutionary hypotheses of multiple LDD syndromes in a biogeographic sense. Nevertheless, our study sheds some light on the importance of evolutionary changes in diaspores over time (speciation). Two recent papers using endemic species did not find loss of dispersability in the Galápagos (Vargas *et al*. 2014) or the Canaries (Vazačová and Münzbergová 2014), as historically proposed for the flora of Hawai’i (Carlquist 1966). Our results revealed that the proportion of species bearing multiple dispersal syndromes is not lower among endemics (adapted to new island conditions) than non-endemic species (more related to mainland colonizers), as predicted by the historical hypothesis of loss of dispersability (see Vargas 2014). Instead, the proportion of multichorous species appears to have been maintained during the process of speciation in the endemic plants of the Azores (5 endemics out of 9 native species), Canaries (8 endemics among 17 native species) and Galápagos (6 endemics among 18 native species). The question remains as to whether the loss of dispersability hypothesis is only restricted to certain archipelagos such as Hawai’i and Samoa (Carlquist 1966). Phylogenetic studies based on sister species and estimates of divergence times are essential to test evolutionary transitions, including loss of dispersability during the speciation process and the two models of evolutionary transition over time.

## Conclusions

The emergence of angiosperms more than 180 million years ago is characterized by the acquisition of three dispersal units (seed, fruit, infructescence) subject to evolutionary change, and thus to favour morphological differentiation into multiple LDD syndromes. Nevertheless, only a few species bear two or more syndromes (<5 % of any flora tested herein). In addition, not all syndrome combinations appear to have been similarly acquired during this long period of time. Some syndrome pairs (e.g. anemochorous/endozoochorous) are absent, whereas others (e.g. anemochorous/thalassochorous) are more common. The floras of Europe, Azores, Canaries and Galápagos also aid us in interpreting that the combination of some diplochorous traits could be the result of both evolutionary and ecological constraints. For instance, endozoochorous/thalassochorous traits appear to be more common in tropical areas, which supports a pattern of latitudinal variation. In a nutshell, our results show that the presence of any dispersal syndrome confers a colonization advantage; however, species having more than one dispersal syndrome possess only a meagre, and not always statistically traceable, improvement in island colonization by plants.

## Sources of Funding

This study is framed within a biogeographic project (CGL2012-C02-01) financed by the *Ministerio de Economía y Competitividad* (Spain). Y.A. was financed by a pre-PhD contract from the *Ministerio de Ciencia y Tecnología* (Spain). R.H.H. was funded by the FCT grant IF/00441/2013 (Portugal) and the Marie Curie Action CIG-321794 (European Union).

## Contributions by the Authors

P.V. designed the study; R.H.H. and Y.A. analysed the data; R.H.H., Y.A., M.N. and P.V. collected the data; P.V. led the writing; all authors read the text and provided significant improvement.

## Conflict of Interest Statement

None declared.
